# Supplementary material for: Changing perception and improving knowledge of leprosy: An intervention study in Uttar Pradesh, India
Source: PLoS Negl Trop Dis. 2021 Aug 23;15(8):e0009654. doi: 10.1371/journal.pntd.0009654 (PMC8412405; doi:10.1371/journal.pntd.0009654)
Supplement: S1 Text — (DOCX) [file pntd.0009654.s001.docx]

**S1 Supporting information file - intervention development based on 6SQuID**

The interventions were developed based on the six steps of quality intervention development (6SQuID):

1. Defining and understanding the problem and its causes
2. Identifying which causal or contextual factors are modifiable: which have the greatest scope for change and who would benefit most;
3. Deciding on the mechanisms of change;
4. Clarifying how these will be delivered;
5. Testing and adapting the intervention; and
6. Collecting sufficient evidence of effectiveness to proceed to a rigorous evaluation.

1. Defining and understanding the problem and its causes
The number of new leprosy patients was 202,185 in 2019 globally. India accounted for 114,451 (57%) of the global number of those new leprosy patients. Inadequate knowledge about leprosy (e.g. unawareness of symptoms and treatment, belief in self-cure), negative beliefs about leprosy among persons affected and health workers and misdiagnosis all contribute to late diagnosis of leprosy [1–3].

We conducted an exploratory study to understand the knowledge, attitudes and practices of leprosy in Fatehpur and Chandauli district [4]. We found high levels of community stigma and low levels of knowledge about leprosy in these areas [4]. This warrants the mounting of effective community education and behavior change interventions about leprosy to (1) optimize early case detection in leprosy; (2) positively influence the perception of leprosy; and (3) reduce the stigma surrounding leprosy. In addition, these interventions should help increase the community acceptance of the PEP++ study and adherence to treatment. An overview of the problem and its causes can be found in Table 1.

Table 1. Step 1: understanding the problem and its causes

| **Aspect** | **Answer** |
| --- | --- |
| **Nature and extent of main problem** | - Stigma levels are high. The mean EMIC-CSS (stigma) score was 15.9. Community members had the highest mean EMIC-CSS score (17.4), followed by health workers (15.0) and close contacts (12.4). The mean SDS (social distance) score was 6.6. Community members had the highest mean SDS scores (7.2), followed by close contacts (6.7) and health workers (3.4). - Knowledge of leprosy is low: 12% of the participants had adequate knowledge of leprosy, 76% had moderate knowledge and 14% had poor knowledge of leprosy. - Knowledge is especially poor regarding early symptoms, cause and mode of transmission. This is the case for persons affected by leprosy, their contacts and community members. Knowledge of health workers is relatively good. - Participants have never heard of the PEP++ project and PEP++ as preventive treatment. |
| **Causes and contributing factors** | - We found three main drivers of stigma:   - Poor knowledge and misconceptions about leprosy (lower knowledge of leprosy was associated with more stigma towards persons affected by leprosy).   - Local beliefs (certain beliefs were found to be associated with more stigma towards persons affected by leprosy).   - Fear of contagion. - Little or no education was associated with higher levels of stigma and social distance. - The image that community members have of persons affected by leprosy is likely not based on knowledge from personal contact, but on incorrect information and negative beliefs. - There were higher levels of stigma among participants who thought that:   - Leprosy is caused by an unclean environment   - Leprosy is a divine punishment for sins   - Leprosy is transmitted through skin contact   - Leprosy is transmitted via air   - Leprosy is contagious or didn’t know if leprosy is contagious or not after treatment   - Said they didn’t know the early symptoms of leprosy |
| **Consequences** | - Social exclusion - Participation restrictions - Late diagnosis and therefore late(r) treatment - Possibly low acceptance of the PEP++ project and its preventive regiments (SDR-PEP and PEP++) |

2 Identifying which causal or contextual factors are modifiable: which have the greatest scope for change and who would benefit most

Table 2. Step 2: modifiable factors

| **Factor** | **Evidence modifiable** |
| --- | --- |
| **Poor knowledge and misconceptions about leprosy** | There have been interventions who have challenged certain misconceptions and poor knowledge and have successfully improved this. In addition, participants with more knowledge about leprosy had lower levels of stigma. |
| **Local beliefs** | Historical change |
| **Fear of contagion** | There have been interventions who have reduced this |

3. Deciding on the mechanisms of change
Deciding on the mechanisms of change was done in several ways. As part of the exploratory study (results presented above) a communication needs assessment was conducted. The purpose of this communication needs assessment was to explore what means of communication people are using and prefer to use in the project areas. This assessment revealed that:

- Radio is not used often.
- TV is popular, but most people do not own a TV.
- 80% of the participants own a mobile phone. Only 9-21% have internet on their phone.
- The participants have on average 10-11 hours of electricity daily.
- Participants think health providers (health care workers, doctors, Accredited Social Health Activists (ASHAs)) and heads of the villages are the most credible persons to receive health-related information from.
- Posters/banners, community group meetings, loudspeakers and street play/theatre are considered to be the most appropriate means of communication in the communities.

The exploratory study was followed by a workshop. Several key persons were involved in the workshop: persons from the intended audiences (persons affected by leprosy, contacts, community members and health workers from the project areas), communication experts, leprosy experts, and staff working at NLR and/or the PEP++ project. During the workshop, results from the exploratory study were presented and participants were asked to brainstorm about appropriate messages and means of communication. This was done for each participant group (in smaller groups). The following means of communication were suggested (Table 3):

Table 3. Means of communication suggested by participants of the workshop.

| **Target group** | **Possible means of communication** |
| --- | --- |
| **Persons affected by leprosy** | - Community/group meetings that are joined by health workers - Peer counselling/education - Brochures with pictures for illiterate people - TV - Wall painting - Interventions that involve school children, the panchayat (local government) and/or volunteers |
| **Close contacts** | - Community/group meetings that are joined by doctors - SMS or WhatsApp messages - A mobile app - A short video - Dialogue and face-to-face “contact with contacts”, door-to-door visits of close contacts who show are ‘living good examples’ (they have contact with persons affected but not infected themselves) - A network of contacts across the villages |
| **Community** | - Community/group meetings that are joined by health workers, social gatherings - Mass media: community-made video shown at village meetings, cinema - Printed media, possible locations could include the panchayat hall, health center, outside temples, schools - Public awareness campaign - Door-to-door visits by ASHAs (community health workers), who could provide information about leprosy |
| **Health care workers** | - Interaction between health workers and persons affected, demonstrate by example (e.g. touching patients, experienced health workers who did not develop leprosy but were ‘exposed’), involving health workers in 'interaction events' - Video - Health workers skills training both in the classroom and on the job (lectures, demonstrations, practice, role play) - Printed material, e.g. a frequently asked questions for health workers - Smartphone app (such as the SkinApp or with a frequently asked questions) - WhatsApp group - Incentive for health workers (such as a good performance award or employee of the month) - Refresher trainings for health staff. |

In addition, several messages were suggested at the workshop (Table 4).

Table 4. Messages proposal at the workshop

| **Topic** | **Messages proposed at workshop** | **Priority area? Based on information from the exploratory study** |
| --- | --- | --- |
| **Transmission** | - Leprosy does not spread by touch / Leprosy is touchable / infection does not occur by touch | Yes, only 4% of the participants know leprosy transmits by air |
| **Cause** | - Leprosy does not happen because of misdeeds/sins | Yes, only 11% of the participants know that bacteria are the cause of leprosy |
| **Early symptoms** | - Leprosy is caused by a bacterium - If you have anesthetic patches, visit the hospital | Yes, only 13% of the participants know that skin patches and a loss of sensation are early symptoms of leprosy |
| **Contagious** | - Leprosy is not highly contagious - Leprosy is not contagious when taking treatment, an ulcer is not infectious | Moderate, 43% of the participants know leprosy is not contagious when on treatment |
| **Disabilities** | - Delays in treatment can lead to deformity | Moderate, 69% of the participants know disabilities can be prevented |
| **Treatment** | - Leprosy is curable by MDT, drugs are freely available - What happens if you don’t complete treatment (person affected) | No, 93% of the participants know leprosy can be treated and 80% know leprosy can be treated by medication. |
| **Other** | - Leprosy patient can lead a normal life | Yes, stigma levels are high. |

4. Clarifying how these will be delivered
It was decided that a ‘task force’ of communication professionals and staff working at NLR would refine the messages (of Table 4) and make a final selection of interventions, based on input from the workshop. Based on several discussions, it was decided to include the following interventions: printed material, community meetings, mobile messages and training for health workers (Table 5).

The following main themes for messages were identified: (1) signs and symptoms, (2) ‘touchability’, (3) cause, (4) curability and disabilities, (5) prevention, (6) role of the community.

Table 5. Final selection of interventions. Only interventions that were mentioned by multiple participant groups were considered.

| **Interventions proposed at workshop** | **Remarks** | **Include?** |
| --- | --- | --- |
| Community/group meetings | Mentioned by all groups, also considered to be the one of the most appropriate means of communication in the communities (communication needs assessment) | Yes |
| Printed material (such as brochures, posters, frequently asked questions) | Mentioned by all groups, also considered to be the one of the most appropriate means of communication in the communities (communication needs assessment). In addition, posters could cover a large area. Pictures need to be included for people who are illiterate. | Yes |
| Interventions at schools | Mentioned by persons affected and community members. It was decided to not focus on this for now. However, if printed materials are included, schools could be one of the locations to put the posters. | No |
| TV and/or a short video | TV is not appropriate, given that most people do not own a TV. In addition, a TV commercial is costly. A video would be appropriate and could be shown at community meetings. | Maybe as part of the community meetings |
| SMS or WhatsApp messages and/or a smartphone app | Smartphone applications are not appropriate, given that most people do not have internet on their phone. It was decided to include SMS and voice messages, since this works on phones without internet. Voice messages are also appropriate for persons who are illiterate. | Yes, SMS and voice messages |
| Health workers training | This was already planned as part of the PEP++ project. | Yes |
| ‘Good examples’ of contact between health workers and/or contacts and persons affected, showing they did not develop leprosy | It was decided to try to merge this idea in the other interventions, but not use this as a separate intervention. | No |

5. Testing and adapting the intervention
Draft prototype posters were developed by the CEBC task force over several meetings, based on the exploratory study and workshop (the information presented above).

Because we wanted to be able to determine the impact of each intervention, it was decided to use a stepped-wedge design. This means one method would be introduced in the communities first, then other methods would be added one-by-one every six months. Before each new method is added, a brief assessment of knowledge and stigma levels was done with a smaller sample. It was decided that the posters would be introduced first (since they have the potential to reach everyone in the community), followed by the mobile messages and community meetings. Health worker training was considered ‘standard’ for the implementation of the PEP++ project.

*Testing of the printed material*
The printed material was pilot tested extensively. A pre-test was conducted among the campaign target audience from August to September 2018, to determine the comprehension of participants, appropriateness and attractiveness of the posters. Each poster was presented to the participants one-by-one, in three different designs. During the pre-test, participants were also asked which design of the poster they preferred and what they thought the best place to display the posters would be. Eight focus group discussions and 40 interviews were conducted in total (four focus group discussions and 20 interviews in each district). The posters were adjusted based on the pre-test.

This pre-test was followed by an operational pilot in October 2018. The operational pilot was conducted to confirm the design and operational processes for the roll out of the printed materials. During the operational pilot, posters were put up in the communities for several weeks. In-depth interviews were conducted with the posters to be tested not visible or close to the participant, this was done to see how many people had seen the posters, to check the visibility of the posters and to check the ability of people to recall core messages. In addition, intercept interviews were conducted. Here, the participants were asked for immediate reactions to material that could be seen from where the interview was conducted. A total of 40 in-depth interviews (20 in each district) and 121 intercept interviews were conducted (61 in Chandauli, 60 in Fatehpur). The posters were adjusted and finalized based on these pilot tests.

The mobile messages will be evaluated in January 2021. Health worker training is implemented from December 2020 onwards and will be evaluated in 2021.

6. Collecting sufficient evidence of effectiveness to proceed to a rigorous evaluation
A follow-up survey was conducted with a random sample of participants, using the same methods as the exploratory study, after the posters had been put up for several months. Details of the evaluation of the printed media and community meetings are presented in the manuscript.

# References

1. Opala J, Boillot F. Leprosy among the Limba: illness and healing in the context of world view. Soc Sci Med 1996;42:3–19.

2. Da Silva Souza C, Bacha JT. Delayed diagnosis of leprosy and the potential role of educational activities in Brazil. Lepr Rev 2003;74:249–58.

3. John AS, Rao OS. Awareness and attitudes towards leprosy in urban slums of Kolkata, India. Indian J Lepr 2009;81:135–40.

4. van‘t Noordende AT, Korfage I, Lisam S, Arif MA, Kumar A, van Brakel WH. The role of perceptions and knowledge of leprosy in the elimination of leprosy: A baseline study in Fatehpur district, northern India. PLoS Negl Trop Dis 2019;13:e0007302.
