# Supplementary material for: Changing perception and improving knowledge of leprosy: An intervention study in Uttar Pradesh, India
Source: PLoS Negl Trop Dis. 2021 Aug 23;15(8):e0009654. doi: 10.1371/journal.pntd.0009654 (PMC8412405; doi:10.1371/journal.pntd.0009654)
Supplement: S2 Text — (DOCX) [file pntd.0009654.s002.docx]

**Supporting information file 2 - Posters and their English translation**

Please note that all logos have been removed.

| **Posters / Hindi** | **English** |
| --- | --- |
| 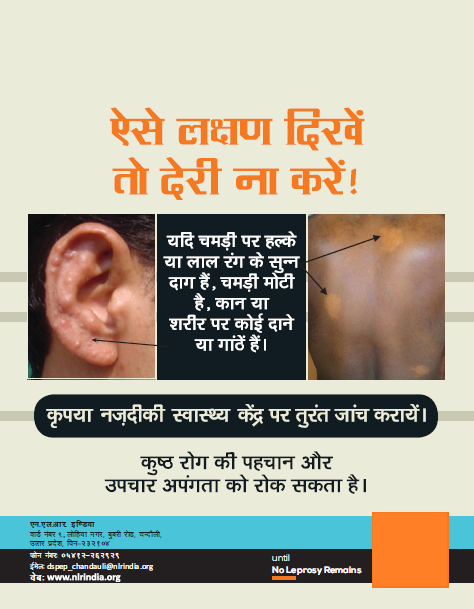 | In case you see any symptoms, then do not delay!  If you have light or red coloured numbness on your skin, tingling in hands and feet, thickened skin, papular or patches on the ears or body  Please investigate immediately at the nearest health centre  Timely identification and treatment can prevent disability |
| 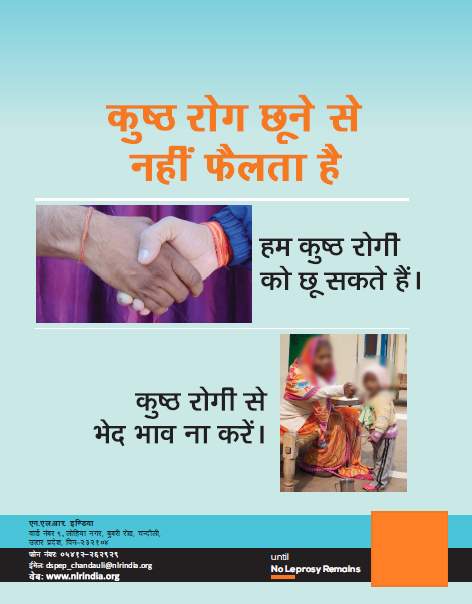 | Leprosy does not spread by touch!  We can touch leprosy patients  Do not discriminate against leprosy |
| 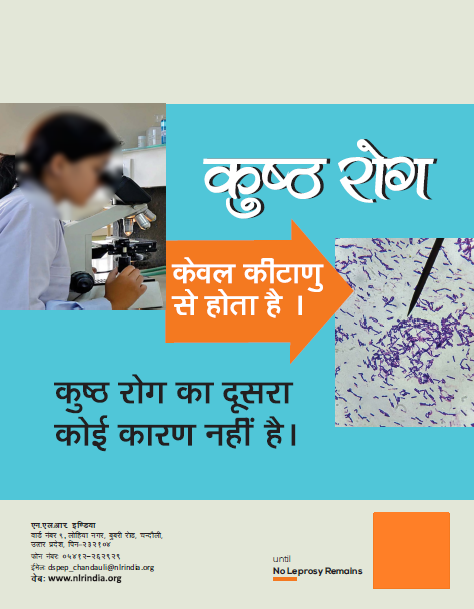 | Leprosy occurs only by germs  There is no other reason for leprosy |
| 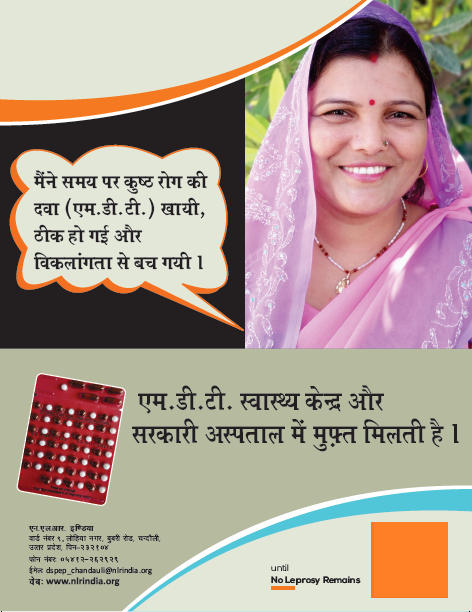 | I have taken MDT for leprosy And now I am completely cured   Persons affected by leprosy can lead a normal life.   I have taken (freely available) MDT on time and did not develop any disabilities. |
| 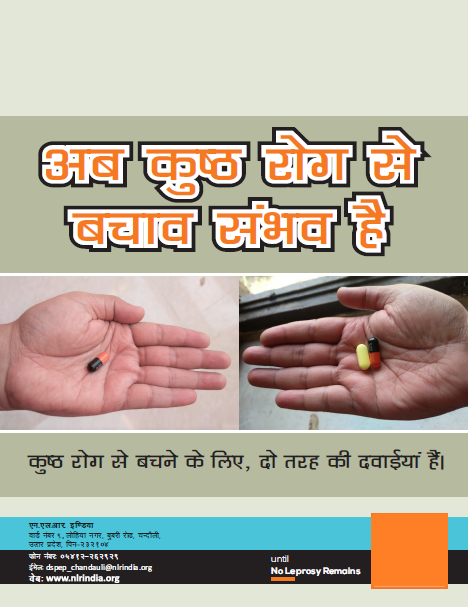 | It is possible to prevent leprosy   To prevent leprosy there are two different drugs |
| 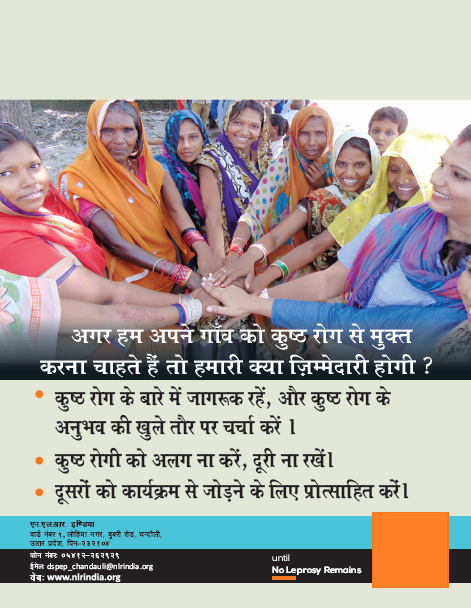 | If we want to make our village leprosy free, what should be our responsibility?   - Be aware of leprosy, and openly discuss leprosy experience - Come! Collaborate in the Leprosy Elimination Programme - Don’t isolate, segregate and don’t keep distance with persons affected by leprosy - Arouse interest of people on leprosy, and participate in leprosy eradication program |
