# Supplementary material for: Changing perception and improving knowledge of leprosy: An intervention study in Uttar Pradesh, India
Source: PLoS Negl Trop Dis. 2021 Aug 23;15(8):e0009654. doi: 10.1371/journal.pntd.0009654 (PMC8412405; doi:10.1371/journal.pntd.0009654)
Supplement: S3 Text — (DOCX) [file pntd.0009654.s003.docx]

**Supporting information file 3 - Quantile regression (0.5 quantile) – questionnaire outcomes Survey 1 and Survey 2**

- Target variable: KAP measure (or SDS or EMIC-CSS)
- Covariates:
  - For the whole dataset: Age, sex, district, education, religion, participant type, data collection period (survey 1 or survey 2)
  - For the dataset per district: Age, sex, education, religion, participant type, data collection period (survey 1 or survey 2)

**Dataset with all participant types
Dataset with both districts
KAP measure**

|  | **Regression coefficient** | **Standard error** | **p-value** | **95% Confidence interval** | |
| --- | --- | --- | --- | --- | --- |
| (Intercept) | 3.500 | .6037 | .000 | 2.316 | 4.684 |
| Age | 2.137E-17 | .0029 | 1.000 | -.006 | .006 |
| From Chandauli district | 2.179E-16 | .0818 | 1.000 | -.160 | .160 |
| Gender, male | .500 | .0868 | .000 | .330 | .670 |
| No (formal) education completed | -.500 | .1267 | .000 | -.748 | -.252 |
| Primary education completed | -2.931E-14 | .1388 | 1.000 | -.272 | .272 |
| Secondary education completed | -1.422E-15 | .1182 | 1.000 | -.232 | .232 |
| Higher education completed | 0^c^ | . | . | . | . |
| Religion, Hinduism | -2.844E-14 | .5882 | 1.000 | -1.154 | 1.154 |
| Religion, Islam | -2.817E-14 | .6058 | 1.000 | -1.188 | 1.188 |
| Religion, Other | 0^c^ | . | . | . | . |
| Person affected by leprosy | 1.000 | .1066 | .000 | .791 | 1.209 |
| Close contact | -8.715E-16 | .1063 | 1.000 | -.209 | .209 |
| Health worker | 1.500 | .1578 | .000 | 1.191 | 1.809 |
| Data collection period (Survey 2) | 1.500 | .0836 | .000 | 1.336 | 1.664 |

**EMIC-CSS**

|  | **Regression coefficient** | **Standard error** | **p-value** | **95% Confidence interval** | |
| --- | --- | --- | --- | --- | --- |
| (Intercept) | 17.000 | 3.0998 | .000 | 10.920 | 23.080 |
| Age | 2.960E-16 | .0171 | 1.000 | -.033 | .033 |
| From Chandauli district | -3.000 | .4712 | .000 | -3.924 | -2.076 |
| Gender, male | -.667 | .4930 | .176 | -1.634 | .300 |
| No (formal) education completed | -.333 | .7372 | .651 | -1.779 | 1.113 |
| Primary education completed | .333 | .8017 | .678 | -1.239 | 1.906 |
| Secondary education completed | -2.466E-14 | .6737 | 1.000 | -1.322 | 1.322 |
| Higher education completed | 0^c^ | . | . | . | . |
| Religion, Hinduism | 3.667 | 2.9977 | .221 | -2.213 | 9.547 |
| Religion, Islam | 3.333 | 3.1125 | .284 | -2.772 | 9.439 |
| Religion, Other | 0^c^ | . | . | . | . |
| Close contact | -4.333 | .5435 | .000 | -5.400 | -3.267 |
| Health worker | -4.333 | .8299 | .000 | -5.961 | -2.705 |
| Data collection period (Survey 2) | -7.333 | .4832 | .000 | -8.281 | -6.386 |

**SDS**

|  | **Regression coefficient** | **Standard error** | **p-value** | **95% Confidence interval** | |
| --- | --- | --- | --- | --- | --- |
| (Intercept) | 2.500 | 1.7437 | .152 | -.920 | 5.920 |
| Age | -6.679E-17 | .0096 | 1.000 | -.019 | .019 |
| From Chandauli district | -1.000 | .2650 | .000 | -1.520 | -.480 |
| Gender, male | -.500 | .2773 | .072 | -1.044 | .044 |
| No (formal) education completed | 1.500 | .4147 | .000 | .687 | 2.313 |
| Primary education completed | 1.500 | .4510 | .001 | .615 | 2.385 |
| Secondary education completed | .500 | .3790 | .187 | -.243 | 1.243 |
| Higher education completed | 0^c^ | . | . | . | . |
| Religion, Hinduism | 4.000 | 1.6862 | .018 | .692 | 7.308 |
| Religion, Islam | 3.000 | 1.7508 | .087 | -.434 | 6.434 |
| Religion, Other | 0^c^ | . | . | . | . |
| Close contact | -1.500 | .3057 | .000 | -2.100 | -.900 |
| Health worker | -3.000 | .4668 | .000 | -3.916 | -2.084 |
| Data collection period (Survey 2) | -2.000 | .2718 | .000 | -2.533 | -1.467 |

**Chandauli district (all participant types)
KAP measure**

|  | **Regression coefficient** | **Standard error** | **p-value** | **95% Confidence interval** | |
| --- | --- | --- | --- | --- | --- |
| (Intercept) | 4.383 | .5694 | .000 | 3.266 | 5.500 |
| Age | 1.122E-6 | .0034 | 1.000 | -.007 | .007 |
| Gender, male | .000 | .1051 | .998 | -.206 | .206 |
| No (formal) education completed | -1.000 | .1507 | .000 | -1.295 | -.704 |
| Primary education completed | .000 | .1629 | .999 | -.320 | .320 |
| Secondary education completed | .000 | .1405 | .999 | -.276 | .275 |
| Higher education completed | 0^c^ | . | . | . | . |
| Religion, Hinduism | -.383 | .5562 | .491 | -1.474 | .708 |
| Religion, Islam | -.383 | .5837 | .512 | -1.528 | .763 |
| Religion, Other | 0^c^ | . | . | . | . |
| Person affected by leprosy | .000 | .1308 | .998 | -.256 | .257 |
| Close contact | -.001 | .1317 | .996 | -.259 | .258 |
| Health worker | 1.000 | .1927 | .000 | .622 | 1.378 |
| Data collection period (Survey 2) | 2.000 | .1037 | .000 | 1.796 | 2.203 |

**EMIC-CSS**

|  | **Regression coefficient** | **Standard error** | **p-value** | **95% Confidence interval** | |
| --- | --- | --- | --- | --- | --- |
| (Intercept) | 12.000 | 2.1985 | .000 | 7.685 | 16.315 |
| Age | 3.713E-17 | .0147 | 1.000 | -.029 | .029 |
| Gender, male | 1.660E-15 | .4408 | 1.000 | -.865 | .865 |
| No (formal) education completed | 1.000 | .6544 | .127 | -.284 | 2.284 |
| Primary education completed | 1.000 | .6984 | .153 | -.371 | 2.371 |
| Secondary education completed | 1.000 | .6020 | .097 | -.182 | 2.182 |
| Higher education completed | 0^c^ | . | . | . | . |
| Religion, Hinduism | 6.000 | 2.1351 | .005 | 1.809 | 10.191 |
| Religion, Islam | 6.000 | 2.2576 | .008 | 1.569 | 10.431 |
| Religion, Other | 0^c^ | . | . | . | . |
| Close contact | -4.000 | .5066 | .000 | -4.994 | -3.006 |
| Health worker | -4.000 | .7606 | .000 | -5.493 | -2.507 |
| Data collection period (Survey 2) | -10.000 | .4502 | .000 | -10.884 | -9.116 |

**SDS**

|  | **Regression coefficient** | **Standard error** | **p-value** | **95% Confidence interval** | |
| --- | --- | --- | --- | --- | --- |
| (Intercept) | 1.000 | 1.0992 | .363 | -1.158 | 3.158 |
| Age | -1.046E-18 | .0073 | 1.000 | -.014 | .014 |
| Gender, male | 1.848E-16 | .2204 | 1.000 | -.433 | .433 |
| No (formal) education completed | 1.000 | .3272 | .002 | .358 | 1.642 |
| Primary education completed | 1.000 | .3492 | .004 | .315 | 1.685 |
| Secondary education completed | 1.000 | .3010 | .001 | .409 | 1.591 |
| Higher education completed | 0^c^ | . | . | . | . |
| Religion, Hinduism | 4.000 | 1.0675 | .000 | 1.905 | 6.095 |
| Religion, Islam | 3.000 | 1.1288 | .008 | .784 | 5.216 |
| Religion, Other | 0^c^ | . | . | . | . |
| Close contact | -1.000 | .2533 | .000 | -1.497 | -.503 |
| Health worker | -3.000 | .3803 | .000 | -3.746 | -2.254 |
| Data collection period (Survey 2) | -2.000 | .2251 | .000 | -2.442 | -1.558 |

**Fatehpur district (all participant types)
KAP measure**

|  | **Regression coefficient** | **Standard error** | **p-value** | **95% Confidence interval** | |
| --- | --- | --- | --- | --- | --- |
| (Intercept) | 2.038 | 2.0093 | .311 | -1.905 | 5.982 |
| Age | .005 | .0052 | .291 | -.005 | .016 |
| Gender, male | .791 | .1470 | .000 | .503 | 1.080 |
| No (formal) education completed | -.533 | .2186 | .015 | -.962 | -.104 |
| Primary education completed | -.374 | .2427 | .124 | -.850 | .103 |
| Secondary education completed | -.220 | .2029 | .279 | -.618 | .178 |
| Higher education completed | 0^c^ | . | . | . | . |
| Religion, Hinduism | 1.033 | 1.9933 | .604 | -2.879 | 4.945 |
| Religion, Islam | 1.088 | 2.0071 | .588 | -2.851 | 5.027 |
| Religion, Other | 0^c^ | . | . | . | . |
| Person affected by leprosy | 1.418 | .1774 | .000 | 1.069 | 1.766 |
| Close contact | .176 | .1758 | .318 | -.169 | .521 |
| Health worker | 1.643 | .2619 | .000 | 1.129 | 2.157 |
| Data collection period (Survey 2) | 1.258 | .1380 | .000 | .987 | 1.529 |

**EMIC-CSS**

|  | **Regression coefficient** | **Standard error** | **p-value** | **95% Confidence interval** | |
| --- | --- | --- | --- | --- | --- |
| (Intercept) | 28.787 | 10.1263 | .005 | 8.903 | 48.671 |
| Age | -.019 | .0307 | .547 | -.079 | .042 |
| Gender, male | -2.102 | .8430 | .013 | -3.757 | -.447 |
| No (formal) education completed | 1.009 | 1.2733 | .428 | -1.491 | 3.509 |
| Primary education completed | 1.454 | 1.4108 | .303 | -1.317 | 4.224 |
| Secondary education completed | .259 | 1.1447 | .821 | -1.989 | 2.507 |
| Higher education completed | 0^c^ | . | . | . | . |
| Religion, Hinduism | -10.463 | 10.0155 | .297 | -30.129 | 9.203 |
| Religion, Islam | -10.759 | 10.1205 | .288 | -30.632 | 9.113 |
| Religion, Other | 0^c^ | . | . | . | . |
| Close contact | -3.028 | .8881 | .001 | -4.772 | -1.284 |
| Health worker | -1.361 | 1.3670 | .320 | -4.045 | 1.323 |
| Data collection period (Survey 2) | -1.741 | .7935 | .029 | -3.299 | -.183 |

**SDS**

|  | **Regression coefficient** | **Standard error** | **p-value** | **95% Confidence interval** | |
| --- | --- | --- | --- | --- | --- |
| (Intercept) | 7.775 | 5.8840 | .187 | -3.778 | 19.329 |
| Age | -.045 | .0178 | .012 | -.080 | -.010 |
| Gender, male | -2.067 | .4898 | .000 | -3.029 | -1.106 |
| No (formal) education completed | 3.876 | .7398 | .000 | 2.424 | 5.329 |
| Primary education completed | 3.169 | .8198 | .000 | 1.559 | 4.778 |
| Secondary education completed | .854 | .6652 | .200 | -.452 | 2.160 |
| Higher education completed | 0^c^ | . | . | . | . |
| Religion, Hinduism | 1.146 | 5.8196 | .844 | -10.281 | 12.573 |
| Religion, Islam | -.056 | 5.8806 | .992 | -11.603 | 11.491 |
| Religion, Other | 0^c^ | . | . | . | . |
| Close contact | -1.685 | .5161 | .001 | -2.699 | -.672 |
| Health worker | -2.315 | .7943 | .004 | -3.874 | -.755 |
| Data collection period (Survey 2) | -2.292 | .4611 | .000 | -3.197 | -1.387 |

**Dataset with only persons affected by leprosy
Dataset with both districts
KAP measure**

|  | **Regression coefficient** | **Standard error** | **p-value** | **95% Confidence interval** | |
| --- | --- | --- | --- | --- | --- |
| (Intercept) | 5.000 | .5999 | .000 | 3.821 | 6.179 |
| Age | 6.594E-18 | .0093 | 1.000 | -.018 | .018 |
| Gender, male | 7.959E-16 | .2878 | 1.000 | -.566 | .566 |
| From Chandauli district | -1.000 | .2602 | .000 | -1.512 | -.488 |
| No (formal) education completed | -1.000 | .3843 | .010 | -1.756 | -.244 |
| Primary education completed | -1.000 | .4330 | .021 | -1.851 | -.149 |
| Secondary education completed | -1.000 | .3884 | .010 | -1.764 | -.236 |
| Higher education completed | 0^c^ | . | . | . | . |
| Religion, Hinduism | 8.157E-17 | .4879 | 1.000 | -.959 | .959 |
| Religion, Islam | 0^c^ | . | . | . | . |
| Data collection period (Survey 2) | 3.000 | .2641 | .000 | 2.481 | 3.519 |

**Chandauli district (persons affected by leprosy)
KAP measure**

|  | **Regression coefficient** | **Standard error** | **p-value** | **95% Confidence interval** | |
| --- | --- | --- | --- | --- | --- |
| (Intercept) | 4.000 | .4320 | .000 | 3.148 | 4.852 |
| Age | -3.123E-17 | .0058 | 1.000 | -.011 | .011 |
| Gender, male | -9.021E-17 | .1862 | 1.000 | -.367 | .367 |
| No (formal) education completed | -1.000 | .2368 | .000 | -1.467 | -.533 |
| Primary education completed | -1.000 | .2647 | .000 | -1.522 | -.478 |
| Secondary education completed | -1.000 | .2309 | .000 | -1.455 | -.545 |
| Higher education completed | 0^c^ | . | . | . | . |
| Religion, Hinduism | -6.731E-16 | .3847 | 1.000 | -.759 | .759 |
| Religion, Islam | 0^c^ | . | . | . | . |
| Data collection period (Survey 2) | 3.000 | .1638 | .000 | 2.677 | 3.323 |

**Fatehpur district (persons affected by leprosy)
KAP measure**

|  | **Regression coefficient** | **Standard error** | **p-value** | **95% Confidence interval** | |
| --- | --- | --- | --- | --- | --- |
| (Intercept) | 4.000 | .6581 | .000 | 2.702 | 5.298 |
| Age | 8.317E-18 | .0114 | 1.000 | -.023 | .023 |
| Gender, male | 5.566E-16 | .3467 | 1.000 | -.684 | .684 |
| No (formal) education completed | -8.757E-16 | .4925 | 1.000 | -.971 | .971 |
| Primary education completed | 1.625E-16 | .5512 | 1.000 | -1.087 | 1.087 |
| Secondary education completed | -2.311E-16 | .5056 | 1.000 | -.997 | .997 |
| Higher education completed | 0^c^ | . | . | . | . |
| Religion, Hinduism | -2.697E-17 | .5187 | 1.000 | -1.023 | 1.023 |
| Religion, Islam | 0^c^ | . | . | . | . |
| Data collection period (Survey 2) | 3.000 | .3300 | .000 | 2.349 | 3.651 |

**Dataset with only close contacts and community members
Dataset with both districts
KAP measure**

|  | **Regression coefficient** | **Standard error** | **p-value** | **95% Confidence interval** | |
| --- | --- | --- | --- | --- | --- |
| (Intercept) | 4.000 | .5678 | .000 | 2.886 | 5.114 |
| Age | 1.397E-17 | .0032 | 1.000 | -.006 | .006 |
| From Chandauli district | 1.538E-15 | .0928 | 1.000 | -.182 | .182 |
| Gender, male | 2.487E-15 | .0972 | 1.000 | -.191 | .191 |
| No (formal) education completed | -1.000 | .1364 | .000 | -1.268 | -.732 |
| Primary education completed | -2.117E-15 | .1478 | 1.000 | -.290 | .290 |
| Secondary education completed | -1.839E-15 | .1258 | 1.000 | -.247 | .247 |
| Higher education completed | 0^c^ | . | . | . | . |
| Religion, Hinduism | -5.073E-16 | .5466 | 1.000 | -1.072 | 1.072 |
| Religion, Islam | 4.264E-17 | .5679 | 1.000 | -1.114 | 1.114 |
| Religion, Other | 0^c^ | . | . | . | . |
| Close contact | -7.700E-16 | .0993 | 1.000 | -.195 | .195 |
| Data collection period (Survey 2) | 1.000 | .0954 | .000 | .813 | 1.187 |

**EMIC-CSS**

|  | **Regression coefficient** | **Standard error** | **p-value** | **95% Confidence interval** | |
| --- | --- | --- | --- | --- | --- |
| (Intercept) | 15.618 | 2.9731 | .000 | 9.785 | 21.451 |
| Age | .018 | .0170 | .284 | -.015 | .051 |
| From Chandauli district | -2.618 | .4857 | .000 | -3.571 | -1.665 |
| Gender, male | -.545 | .5088 | .284 | -1.544 | .453 |
| No (formal) education completed | -.255 | .7140 | .722 | -1.655 | 1.146 |
| Primary education completed | .473 | .7741 | .542 | -1.046 | 1.991 |
| Secondary education completed | .327 | .6585 | .619 | -.965 | 1.619 |
| Higher education completed | 0^c^ | . | . | . | . |
| Religion, Hinduism | 3.600 | 2.8622 | .209 | -2.015 | 9.215 |
| Religion, Islam | 3.345 | 2.9736 | .261 | -2.488 | 9.179 |
| Religion, Other | 0^c^ | . | . | . | . |
| Close contact | -4.073 | .5200 | .000 | -5.093 | -3.053 |
| Data collection period (Survey 2) | -7.255 | .4996 | .000 | -8.235 | -6.274 |

**SDS**

|  | **Regression coefficient** | **Standard error** | **p-value** | **95% Confidence interval** | |
| --- | --- | --- | --- | --- | --- |
| (Intercept) | 2.920 | 1.8491 | .115 | -.708 | 6.547 |
| Age | -.012 | .0106 | .260 | -.033 | .009 |
| From Chandauli district | -1.292 | .3021 | .000 | -1.885 | -.700 |
| Gender, male | -.745 | .3165 | .019 | -1.366 | -.124 |
| No (formal) education completed | 2.235 | .4441 | .000 | 1.364 | 3.106 |
| Primary education completed | 2.075 | .4815 | .000 | 1.130 | 3.019 |
| Secondary education completed | .813 | .4095 | .047 | .010 | 1.616 |
| Higher education completed | 0^c^ | . | . | . | . |
| Religion, Hinduism | 4.245 | 1.7801 | .017 | .753 | 7.737 |
| Religion, Islam | 3.161 | 1.8494 | .088 | -.467 | 6.790 |
| Religion, Other | 0^c^ | . | . | . | . |
| Close contact | -1.179 | .3234 | .000 | -1.813 | -.544 |
| Data collection period (Survey 2) | -2.667 | .3107 | .000 | -3.276 | -2.057 |

**Chandauli district (close contacts and community members)**

**KAP measure**

|  | **Regression coefficient** | **Standard error** | **p-value** | **95% Confidence interval** | |
| --- | --- | --- | --- | --- | --- |
| (Intercept) | 5.000 | .5374 | .000 | 3.945 | 6.055 |
| Age | 1.123E-17 | .0037 | 1.000 | -.007 | .007 |
| Gender, male | 2.762E-16 | .1142 | 1.000 | -.224 | .224 |
| No (formal) education completed | -1.000 | .1609 | .000 | -1.316 | -.684 |
| Primary education completed | -2.795E-16 | .1711 | 1.000 | -.336 | .336 |
| Secondary education completed | -1.943E-16 | .1488 | 1.000 | -.292 | .292 |
| Higher education completed | 0^c^ | . | . | . | . |
| Religion, Hinduism | -1.000 | .5207 | .055 | -2.022 | .022 |
| Religion, Islam | -1.000 | .5510 | .070 | -2.082 | .082 |
| Religion, Other | 0^c^ | . | . | . | . |
| Close contact | -6.000E-16 | .1237 | 1.000 | -.243 | .243 |
| Data collection period (Survey 2) | 2.000 | .1182 | .000 | 1.768 | 2.232 |

**EMIC-CSS**

|  | **Regression coefficient** | **Standard error** | **p-value** | **95% Confidence interval** | |
| --- | --- | --- | --- | --- | --- |
| (Intercept) | 11.000 | 2.1496 | .000 | 6.780 | 15.220 |
| Age | 2.648E-16 | .0148 | 1.000 | -.029 | .029 |
| Gender, male | 1.000 | .4570 | .029 | .103 | 1.897 |
| No (formal) education completed | 1.000 | .6435 | .121 | -.263 | 2.263 |
| Primary education completed | -2.051E-15 | .6844 | 1.000 | -1.344 | 1.344 |
| Secondary education completed | -8.880E-16 | .5953 | 1.000 | -1.169 | 1.169 |
| Higher education completed | 0^c^ | . | . | . | . |
| Religion, Hinduism | 7.000 | 2.0829 | .001 | 2.911 | 11.089 |
| Religion, Islam | 6.000 | 2.2039 | .007 | 1.673 | 10.327 |
| Religion, Other | 0^c^ | . | . | . | . |
| Close contact | -4.000 | .4949 | .000 | -4.972 | -3.028 |
| Data collection period (Survey 2) | -10.000 | .4728 | .000 | -10.928 | -9.072 |

**SDS**

|  | **Regression coefficient** | **Standard error** | **p-value** | **95% Confidence interval** | |
| --- | --- | --- | --- | --- | --- |
| (Intercept) | 2.000 | 1.6122 | .215 | -1.165 | 5.165 |
| Age | 2.027E-17 | .0111 | 1.000 | -.022 | .022 |
| Gender, male | -8.330E-16 | .3427 | 1.000 | -.673 | .673 |
| No (formal) education completed | 1.000 | .4827 | .039 | .052 | 1.948 |
| Primary education completed | 1.000 | .5133 | .052 | -.008 | 2.008 |
| Secondary education completed | 1.656E-15 | .4465 | 1.000 | -.877 | .877 |
| Higher education completed | 0^c^ | . | . | . | . |
| Religion, Hinduism | 4.000 | 1.5622 | .011 | .933 | 7.067 |
| Religion, Islam | 2.000 | 1.6529 | .227 | -1.245 | 5.245 |
| Religion, Other | 0^c^ | . | . | . | . |
| Close contact | -1.000 | .3712 | .007 | -1.729 | -.271 |
| Data collection period (Survey 2) | -3.000 | .3546 | .000 | -3.696 | -2.304 |

**Fatehpur district (close contacts and community members)
KAP measure**

|  | **Regression coefficient** | **Standard error** | **p-value** | **95% Confidence interval** | |
| --- | --- | --- | --- | --- | --- |
| (Intercept) | 2.000 | 2.8056 | .476 | -3.511 | 7.511 |
| Age | 3.401E-17 | .0090 | 1.000 | -.018 | .018 |
| Gender, male | 1.000 | .2583 | .000 | .493 | 1.507 |
| No (formal) education completed | -3.673E-15 | .3601 | 1.000 | -.707 | .707 |
| Primary education completed | -1.326E-15 | .3993 | 1.000 | -.784 | .784 |
| Secondary education completed | -8.287E-16 | .3289 | 1.000 | -.646 | .646 |
| Higher education completed | 0^c^ | . | . | . | . |
| Religion, Hinduism | 1.000 | 2.7717 | .718 | -4.444 | 6.444 |
| Religion, Islam | 1.000 | 2.8017 | .721 | -4.503 | 6.503 |
| Religion, Other | 0^c^ | . | . | . | . |
| Close contact | 4.334E-16 | .2463 | 1.000 | -.484 | .484 |
| Data collection period (Survey 2) | 1.000 | .2403 | .000 | .528 | 1.472 |

**EMIC-CSS**

|  | **Regression coefficient** | **Standard error** | **p-value** | **95% Confidence interval** | |
| --- | --- | --- | --- | --- | --- |
| (Intercept) | 29.000 | 9.8197 | .003 | 9.712 | 48.288 |
| Age | -1.743E-16 | .0316 | 1.000 | -.062 | .062 |
| Gender, male | -3.000 | .9039 | .001 | -4.775 | -1.225 |
| No (formal) education completed | 1.000 | 1.2602 | .428 | -1.475 | 3.475 |
| Primary education completed | 2.000 | 1.3974 | .153 | -.745 | 4.745 |
| Secondary education completed | 1.000 | 1.1512 | .385 | -1.261 | 3.261 |
| Higher education completed | 0^c^ | . | . | . | . |
| Religion, Hinduism | -11.000 | 9.7011 | .257 | -30.055 | 8.055 |
| Religion, Islam | -11.000 | 9.8058 | .262 | -30.261 | 8.261 |
| Religion, Other | 0^c^ | . | . | . | . |
| Close contact | -3.000 | .8621 | .001 | -4.693 | -1.307 |
| Data collection period (Survey 2) | -2.000 | .8410 | .018 | -3.652 | -.348 |

**SDS**

|  | **Regression coefficient** | **Standard error** | **p-value** | **95% Confidence interval** | |
| --- | --- | --- | --- | --- | --- |
| (Intercept) | 8.143 | 6.4797 | .209 | -4.585 | 20.870 |
| Age | -.048 | .0208 | .023 | -.089 | -.007 |
| Gender, male | -2.381 | .5964 | .000 | -3.553 | -1.209 |
| No (formal) education completed | 4.048 | .8316 | .000 | 2.414 | 5.681 |
| Primary education completed | 3.333 | .9221 | .000 | 1.522 | 5.145 |
| Secondary education completed | 1.238 | .7597 | .104 | -.254 | 2.730 |
| Higher education completed | 0^c^ | . | . | . | . |
| Religion, Hinduism | 1.048 | 6.4014 | .870 | -11.526 | 13.621 |
| Religion, Islam | -.190 | 6.4705 | .977 | -12.900 | 12.519 |
| Religion, Other | 0^c^ | . | . | . | . |
| Close contact | -1.810 | .5689 | .002 | -2.927 | -.692 |
| Data collection period (Survey 2) | -2.333 | .5550 | .000 | -3.423 | -1.243 |

**Dataset with only health workers
Dataset with both districts
KAP measure**

|  | **Regression coefficient** | **Standard error** | **p-value** | **95% Confidence interval** | |
| --- | --- | --- | --- | --- | --- |
| (Intercept) | 3.000 | .8563 | .001 | 1.311 | 4.689 |
| Age | -2.839E-18 | .0076 | 1.000 | -.015 | .015 |
| Gender, male | 1.000 | .1715 | .000 | .662 | 1.338 |
| From Chandauli district | 2.801E-16 | .1598 | 1.000 | -.315 | .315 |
| Primary education completed | 9.992E-16 | 1.1282 | 1.000 | -2.225 | 2.225 |
| Secondary education completed | -1.000 | .4060 | .015 | -1.801 | -.199 |
| Higher education completed | 0^c^ | . | . | . | . |
| Religion, Hinduism | 2.000 | .7953 | .013 | .431 | 3.569 |
| Religion, Islam | 0^c^ | . | . | . | . |
| Data collection period (Survey 2) | 1.000 | .1642 | .000 | .676 | 1.324 |

**EMIC-CSS**

|  | **Regression coefficient** | **Standard error** | **p-value** | **95% Confidence interval** | |
| --- | --- | --- | --- | --- | --- |
| (Intercept) | 13.793 | 5.8462 | .019 | 2.262 | 25.324 |
| Age | -.103 | .0518 | .047 | -.206 | -.001 |
| Gender, male | -2.414 | 1.1706 | .041 | -4.723 | -.105 |
| From Chandauli district | -6.759 | 1.0907 | .000 | -8.910 | -4.607 |
| Primary education completed | -10.034 | 7.7026 | .194 | -25.227 | 5.158 |
| Secondary education completed | -1.586 | 2.7718 | .568 | -7.053 | 3.881 |
| Higher education completed | 0^c^ | . | . | . | . |
| Religion, Hinduism | 8.828 | 5.4302 | .106 | -1.883 | 19.538 |
| Religion, Islam | 0^c^ | . | . | . | . |
| Data collection period (Survey 2) | -4.345 | 1.1208 | .000 | -6.555 | -2.134 |

**SDS**

|  | **Regression coefficient** | **Standard error** | **p-value** | **95% Confidence interval** | |
| --- | --- | --- | --- | --- | --- |
| (Intercept) | 1.000 | 2.5688 | .697 | -4.067 | 6.067 |
| Age | 7.669E-18 | .0228 | 1.000 | -.045 | .045 |
| Gender, male | -1.000 | .5144 | .053 | -2.015 | .015 |
| From Chandauli district | -1.000 | .4793 | .038 | -1.945 | -.055 |
| Primary education completed | -2.000 | 3.3845 | .555 | -8.676 | 4.676 |
| Secondary education completed | 1.000 | 1.2179 | .413 | -1.402 | 3.402 |
| Higher education completed | 0^c^ | . | . | . | . |
| Religion, Hinduism | 2.000 | 2.3860 | .403 | -2.706 | 6.706 |
| Religion, Islam | 0^c^ | . | . | . | . |
| Data collection period (Survey 2) | -1.000 | .4925 | .044 | -1.971 | -.029 |

**Chandauli district (health workers)
KAP measure**

|  | **Regression coefficient** | **Standard error** | **p-value** | **95% Confidence interval** | |
| --- | --- | --- | --- | --- | --- |
| (Intercept) | 5.000 | 1.0381 | .000 | 2.939 | 7.061 |
| Age | 1.933E-17 | .0101 | 1.000 | -.020 | .020 |
| Gender, male | 2.000 | .2454 | .000 | 1.513 | 2.487 |
| Secondary education completed | 2.000 | .6054 | .001 | .798 | 3.202 |
| Higher education completed | 0^c^ | . | . | . | . |
| Religion, Hinduism | -2.458E-16 | .9870 | 1.000 | -1.960 | 1.960 |
| Religion, Islam | 0^c^ | . | . | . | . |
| Data collection period (Survey 2) | 3.727E-16 | .2206 | 1.000 | -.438 | .438 |

**EMIC-CSS**

|  | **Regression coefficient** | **Standard error** | **p-value** | **95% Confidence interval** | |
| --- | --- | --- | --- | --- | --- |
| (Intercept) | 19.100 | 7.2664 | .010 | 4.672 | 33.528 |
| Age | -.050 | .0710 | .483 | -.191 | .091 |
| Gender, male | -3.050 | 1.7181 | .079 | -6.461 | .361 |
| Secondary education completed | -.450 | 4.2376 | .916 | -8.864 | 7.964 |
| Higher education completed | 0^c^ | . | . | . | . |
| Religion, Hinduism | -.450 | 6.9092 | .948 | -14.168 | 13.268 |
| Religion, Islam | 0^c^ | . | . | . | . |
| Data collection period (Survey 2) | -10.300 | 1.5444 | .000 | -13.366 | -7.234 |

**SDS**

|  | **Regression coefficient** | **Standard error** | **p-value** | **95% Confidence interval** | |
| --- | --- | --- | --- | --- | --- |
| (Intercept) | 1.000 | 1.0381 | .338 | -1.061 | 3.061 |
| Age | 1.542E-18 | .0101 | 1.000 | -.020 | .020 |
| Gender, male | -1.000 | .2454 | .000 | -1.487 | -.513 |
| Secondary education completed | 1.000 | .6054 | .102 | -.202 | 2.202 |
| Higher education completed | 0^c^ | . | . | . | . |
| Religion, Hinduism | 6.014E-17 | .9870 | 1.000 | -1.960 | 1.960 |
| Religion, Islam | 0^c^ | . | . | . | . |
| Data collection period (Survey 2) | .000 | .2206 | 1.000 | -.438 | .438 |

**Fatehpur district (health workers)
KAP measure**

|  | **Regression coefficient** | **Standard error** | **p-value** | **95% Confidence interval** | |
| --- | --- | --- | --- | --- | --- |
| (Intercept) | 3.000 | 2.1831 | .173 | -1.335 | 7.335 |
| Age | -1.014E-17 | .0183 | 1.000 | -.036 | .036 |
| Gender, male | 1.000 | .3970 | .013 | .212 | 1.788 |
| Primary education completed | 4.868E-16 | 1.9728 | 1.000 | -3.918 | 3.918 |
| Secondary education completed | -1.000 | .9016 | .270 | -2.790 | .790 |
| Higher education completed | 0^c^ | . | . | . | . |
| Religion, Hinduism | 2.000 | 1.9828 | .316 | -1.938 | 5.938 |
| Religion, Islam | 0^c^ | . | . | . | . |
| Data collection period (Survey 2) | 1.000 | .4008 | .014 | .204 | 1.796 |

**EMIC-CSS**

|  | **Regression coefficient** | **Standard error** | **p-value** | **95% Confidence interval** | |
| --- | --- | --- | --- | --- | --- |
| (Intercept) | 13.471 | 9.6314 | .165 | -5.655 | 32.597 |
| Age | -.118 | .0806 | .148 | -.278 | .042 |
| Gender, male | -1.353 | 1.7514 | .442 | -4.831 | 2.125 |
| Primary education completed | -10.176 | 8.7037 | .245 | -27.460 | 7.107 |
| Secondary education completed | .294 | 3.9775 | .941 | -7.604 | 8.193 |
| Higher education completed | 0^c^ | . | . | . | . |
| Religion, Hinduism | 7.176 | 8.7478 | .414 | -10.195 | 24.548 |
| Religion, Islam | 0^c^ | . | . | . | . |
| Data collection period (Survey 2) | -1.647 | 1.7684 | .354 | -5.159 | 1.865 |

**SDS**

|  | **Regression coefficient** | **Standard error** | **p-value** | **95% Confidence interval** | |
| --- | --- | --- | --- | --- | --- |
| (Intercept) | 3.952 | 4.1063 | .338 | -4.202 | 12.107 |
| Age | -.048 | .0344 | .169 | -.116 | .021 |
| Gender, male | -1.476 | .7467 | .051 | -2.959 | .007 |
| Primary education completed | -1.952 | 3.7108 | .600 | -9.321 | 5.417 |
| Secondary education completed | .333 | 1.6958 | .845 | -3.034 | 3.701 |
| Higher education completed | 0^c^ | . | . | . | . |
| Religion, Hinduism | 2.810 | 3.7296 | .453 | -4.597 | 10.216 |
| Religion, Islam | 0^c^ | . | . | . | . |
| Data collection period (Survey 2) | -2.857 | .7540 | .000 | -4.354 | -1.360 |
