# Supplementary material for: Changing perception and improving knowledge of leprosy: An intervention study in Uttar Pradesh, India
Source: PLoS Negl Trop Dis. 2021 Aug 23;15(8):e0009654. doi: 10.1371/journal.pntd.0009654 (PMC8412405; doi:10.1371/journal.pntd.0009654)
Supplement: S4 Text — (DOCX) [file pntd.0009654.s004.docx]

**Supporting information file 4 - determinants of leprosy knowledge and community stigma for Survey 1 and Survey 2

Please note that the models shown are for the dataset that includes persons affected by leprosy, close contacts and community members.**

- Variables included in the models for Survey 1: participant type, district, age, gender, education, occupation, and for the EMIC-CSS and SDS also ‘KAP score’ and ‘knowing someone affected by leprosy.’
- Variables included in the models for Survey 2: participant type, district, age, gender, education, occupation, marital status, monthly household income, caste, having seen PEP++ posters, and for the EMIC-CSS and SDS also ‘KAP score’ and ‘knowing someone affected by leprosy.’

**Correlations between level of knowledge (KAP measure, in the presence of incorrect answers) about leprosy and the other variables in the dataset

Model for Survey 1 (before the interventions)**

|  | Regression coefficient | Standard error | p-value |
| --- | --- | --- | --- |
| *(Constant)* | 3.867 | .098 | .000 |
| Person affect by leprosy* | .162 | .115 | .162 |
| Close contact* | -.265 | .114 | .020 |
| From Chandauli district* | -.181 | .092 | .049 |
| Not completed any (formal) education | -.392 | .110 | .000 |
| Completed higher education | .338 | .106 | .001 |

* Included in the model to control for confounding. ‘Community members’ are the reference category.
R^2=0.054

**Model for Survey 2 (after the interventions; this is a non-normal distribution, we conducted bootstrapping to correct for non-normality)**

|  | Regression coefficient | Standard error | p-value |
| --- | --- | --- | --- |
| *(Constant)* | 4.528 | .152 | .001 |
| Person affect by leprosy* | 1.656 | .117 | .001 |
| Close contact* | -.072 | .137 | .602 |
| From Chandauli district* | .150 | .110 | .171 |
| Not completed any (formal) education | -.367 | .124 | .005 |
| Completed higher education | .329 | .159 | .041 |
| Monthly household income ≤1,000 INR | -.918 | .153 | .001 |
| Monthly household income 1000-5000 INR | -.263 | .122 | .028 |
| Number of posters seen | .231 | .026 | .001 |

* Included in the model to control for confounding. ‘Community members’ are the reference category.
R^2=0.355

**Correlations between level of stigma (EMIC-CSS) and the other variables in the dataset (this is a non-normal distribution, we conducted bootstrapping to correct for non-normality)

Model for Survey 1 (before the interventions)**

|  | Regression coefficient | Standard error | p-value |
| --- | --- | --- | --- |
| *(Constant)* | 12.257 | .528 | .001 |
| Community member* | 4.930 | .594 | .001 |
| From Chandauli district* | .319 | .505 | .529 |

* Included in the model to control for confounding.
R^2=0.105

**Model for Survey 2 (after the interventions)**

|  | Regression coefficient | Standard error | p-value |
| --- | --- | --- | --- |
| *(Constant)* | 14.823 | .877 | .001 |
| Community member* | 2.114 | .524 | .001 |
| From Chandauli district* | -5.377 | .502 | .001 |
| Completed primary education | 1.446 | .605 | .023 |
| Monthly household income ≤1,000 INR | -2.790 | .623 | .001 |
| Number of posters seen | -.359 | .122 | .005 |
| Total knowledge (KAP) score | -.437 | .152 | .004 |

* Included in the model to control for confounding.
R^2=0.292

**Correlations between level of social distance (SDS) and the other variables in the dataset (this is a non-normal distribution, we conducted bootstrapping to correct for non-normality)
Model for Survey 1 (before the interventions)**

|  | Regression coefficient | Standard error | p-value |
| --- | --- | --- | --- |
| *(Constant)* | 8.038 | .747 | .001 |
| Community member* | 1.206 | .456 | .007 |
| From Chandauli district* | -1.211 | .410 | .004 |
| Male gender | -.854 | .401 | .035 |
| Not completed any (formal) education | 1.516 | .470 | .003 |
| Total knowledge (KAP) score | -.272 | .142 | .047 |

* Included in the model to control for confounding.
R^2=0.050

**Model for Survey 2 (after the interventions)**

|  | Regression coefficient | Standard error | p-value |
| --- | --- | --- | --- |
| *(Constant)* | 7.348 | .776 | .001 |
| Community member* | 1.249 | .361 | .001 |
| From Chandauli district* | -2.451 | .371 | .001 |
| Not completed any (formal) education | 1.092 | .464 | .019 |
| Completed primary education | 1.744 | .505 | .001 |
| Occupation paid work | -1.347 | .405 | .002 |
| Monthly household income 5,001-10,000 INR | 1.698 | .393 | .001 |
| Number of posters seen | -.268 | .082 | .002 |
| Total knowledge (KAP) score | -.516 | .131 | .001 |

* Included in the model to control for confounding.
R^2=0.232
