# Supplementary material for: Changing perception and improving knowledge of leprosy: An intervention study in Uttar Pradesh, India
Source: PLoS Negl Trop Dis. 2021 Aug 23;15(8):e0009654. doi: 10.1371/journal.pntd.0009654 (PMC8412405; doi:10.1371/journal.pntd.0009654)
Supplement: Attachment — Submitted filename: 210614 Point-by-point reply CEBC India.docx [file pntd.0009654.s006.docx]

**Point-by-point reply document**

**Manuscript**: Changing perception and improving knowledge of leprosy: an intervention study in Uttar Pradesh, India (PNTD-D-21-00531)
**Deadline** to submit revised manuscript: mid-July 2021

| **Reviewers’ comments:** | **Response authors** | **Changes in manuscript (highlighted in yellow)** |
| --- | --- | --- |
| Reviewer #1: Introduction:  Provide definitions and explain the difference between these two concepts-perception & knowledge used in the title, also ‘attitudes’  And Introduction: Provide definitions and explain the difference between these concepts in the title and used in the manuscript -perception, knowledge, attitudes and practices | This has been revised. | The following has been added to the introduction: “Definitions Perception comprises knowledge, beliefs, attitudes and emotions. Beliefs link an object (such as a person, group of people, disease, institution or behaviour) to an attribute. For example the belief “leprosy is dangerous”, links “leprosy” (object) to “dangerous” (attribute) [16]. Knowledge refers to theoretical or practical understanding of a subject (facts, skills or objects). Truth and belief are a prerequisite for possessing knowledge: one has a belief in something, and that belief must be true (based on observable and measurable evidence). For example, if you know that leprosy is an infectious disease, then you must believe this, and your belief must be true [17]. An attitude refers to a person's feelings toward and evaluation of an aspect of the person's world, for example an object, person, event, or towards performing specific behaviours [16,18]. It refers to “a person’s location on a dimension of affect or evaluation” and falls on a continuum from very favourable to very unfavourable [16]. Emotions are inner states such as anger, joy, fear or love. Emotions can be consciously experienced, but can also be repressed, inhibited or unconscious [21].” |
| Reviewer #1: Methodology: Organize the text in the following order:  Topic: Changing perception and improving knowledge of leprosy: an intervention study in Uttar Pradesh, India may be modified how the best it suits.  Research design:   - Study phases: Details Baseline(pre- interventional), intervention, Evaluation(post- interventional) - Input and outcome measures | We thank the reviewer for his/her comments. The headings in our methods section comply with those of the SPIRIT checklist (<http://www.spirit-statement.org/wp-content/uploads/2013/01/SPIRIT-Checklist-download-8Jan13.pdf>). We have now clarified that the post-intervention survey (Survey 2) was the evaluation survey. | The following has been added to methods > participant timeline: “Survey 1 was conducted between March 2017 and December 2018. The outcomes represent the pre-intervention (baseline) information. After finalization of the interventions, dissemination of posters started from October 2019 and onwards, and the community meetings were held between September 2019 and February 2020. The post-intervention or evaluation study (Survey 2) was conducted between March and June 2020.”  We have removed “We applied a pre/post intervention study” from the introduction and added a heading “study design” to the methods section: “Study design We applied a pre/post intervention study. The effect of the interventions was evaluated using mixed methods.” |
| Reviewer #1: Study area: The study was conducted in two districts in Uttar Pradesh, northern India: Chandauli (population 1.95 million) and Fatehpur (population 2.63 million). All this population and districts were divided into how many cities, what are Interventional and control areas (with out control area the change can not be attributed to this intervention) *and* Reviewer #1: In case the data represent also certain blocks(similar) from the study area where intervention was not implemented at all, that can be the control area and changes in the interdental area are significantly higher than in the control area, the change will scientifically attributed our KAP intervention. *and* Reviewer #1: The statistical analysis does not measure the change due to the intervention because no control area is mention in the desion. *and* Reviewer #1: The results  can pre presented as a survey findings,  cant be presented as a change of an intervention as no control area data is presented | We thank the reviewer for his/her comments and understand the concern s/he has regarding the lack of a control group. We applied a pre/post intervention study design. We have added a heading to the methods section to clarify this. We have acknowledged this as a limitation also, kindly refer to the final paragraph of the discussion (before the conclusion): “This study has several limitations. First, a randomized controlled design was not feasible given the nature of our intervention, namely, community-wide poster dissemination and meetings.” We have now expanded this section to emphasize it more. While we did not use a randomized controlled design, we did used a random sample of participant pre and post intervention. We believe this is a strength, given that administering a questionnaire can be an intervention by itself (this is not an issue when a random sample is selected). We have added a discussion about attribution versus contribution to our discussion section. | We have removed “We applied a pre/post intervention study” from the introduction and added a heading “study design” to the methods section: “Study design We applied a pre/post intervention study design. The effect of the interventions was evaluated using mixed methods.”  The following has been added to the discussion > methodological considerations: “This study has several limitations. First, a randomized controlled design was not feasible given the nature of our intervention, namely, community-wide poster dissemination and meetings. Instead, we used a pre/post intervention design. It is possible that not all of the observed changes are due to the interventions – some changes may have been caused by other factors. We tried to minimize this by selecting a random sample. However, we cannot rule out other factors that may have contributed to achieve the outcomes. While it is unclear how much of the effect found can be attributed to the interventions, given our findings it is very likely that the interventions have contributed to the outcomes.” |
| Reviewer #1: Duration of the study- For each phase in each cite Study population: (it seems study population includes health care workers, community members, leprosy affected and close contacts) . Need to explain who are these close contacts or family members | The duration of each intervention can be found in Table 1, “Time period disseminated” – Posters: October 2019 - April 2021 (ongoing for full project duration); Meetings: December 2019 -February 2020. Target groups for each intervention are also listed in Table 1.  A description of close contacts has been added. | Methods > eligibility criteria: “We included four groups as participants in the study: (…) (2) close contacts of persons affected by leprosy, these comprised household contacts, family members, neighbours and other social contacts;” |
| Reviewer #1: Data collection methods: For each of the above categories, what is Sample size, Sampling procedure used to decide sample size, methods of data collection, method of recruiting the respondents.  *and* Reviewer #1: Sampling procedure is not explained.  Two districts Chandauli (population 1.95 million) and Fatehpur (population 2.63 million)are studied.  How arrived at total of 1067 participants in   the pre and 843 at post intervention surveys is to be clearly explain | This has been revised. | The following has been added to methods > sample size: “We aimed to include a random sample of at least 100 persons of each target group per district for the interview-administered questionnaires. ~~A detailed description of the sample size calculation can be found elsewhere [23].~~ This is based on an assumed prevalence of, for example, ‘negative attitudes’ of 50% at baseline and wanting to be able to detect a reduction of 20% or more (meaning that the prevalence of negative attitudes in the second survey is 30% or less). Using these parameters, a significance level of 0.05 and a power of 80%, 93 participants are needed before and 93 are needed after the intervention is implemented (calculated using Epi Info StatCalc for cross-sectional studies). To compensate for records that may not be usable or loss to follow up, we aimed to include at least 100 participants per target group. The data of community members for Survey 1 were collected in a separate study but using the same instruments, in the same area and timeframe. Thus, these data were included instead interviewing another sample of community members.” |
| Reviewer #1: Methodology for intervention: What is the total no of villages in the two districts. Posters covered in 606 villages across the two districts, whether covered all the villages, or any criterial to select; Similarly 271 group meeting in how many villages and what is the criteria. | This has been revised. | This has been added to methods > study setting: “The study was conducted in two districts in Uttar Pradesh, northern India: Chandauli (population 1.95 million, 1548 villages) and Fatehpur (population 2.63 million, 1476 villages).”  The following has been added to methods >  “The villages in which the posters were put up were selected based on the number of leprosy patients registered at the health centre since April 2014. Only villages in which at least two patients were registered were selected.” and “The community meetings were held in villages selected from the list of 606 villages in which posters were put up. Villages in which the Pradhan (village leader) was available on scheduled meeting days and where the prior relationship with the Pradhan was good, were selected. Community members were invited to attend the meeting through the Pradhan and by door-to-door visits from community health workers (ASHAs).(…)” |
| Reviewer #1: Data collection was done from a total 1067 participants in the first survey and 843 participants including from all the categories of responding. Need to clarify whether this data collection was done only from the areas ( villages/blocks) where interventions were done; if so the differences in the studied variables between pre and post intervention assessments can not be attributed to the result of this intervention. Such changes may be taking place every where over a period of time due to any other factors. | This has been clarified. | Methods > data collection: “In Survey 2, additional demographic information was collected from the participants about income and caste. In addition, Survey 2 data were collected in the areas in which interventions were conducted.” |
| Reviewer #1: Clarify what is adequate knowledge and cut of points used for other variable measures | We thank the reviewer for his/her comment. Adequate knowledge and other cut-offs have been described in the methods under ‘outcomes’: “The KAP measure was used to assess the knowledge, attitudes and practices of participants regarding leprosy. On some of the questions, multiple answers are possible. A maximum score of eight could be obtained on the KAP if all correct answers are provided, even if incorrect answers were present. We defined ‘poor knowledge’ as a score of two or less out of eight, ‘moderate knowledge’ as a score between two and six and ‘adequate knowledge’ as a score of six or more on the KAP. (…) We operationalized stigmatizing community attitudes towards leprosy as a sum score of 8 or higher on the EMIC-CSS, using the cut-off point of 8 that was proposed by Sermrittirong and colleagues [1]. (…)The SDS has been translated to Hindi and was partially validated among community members of persons affected by leprosy in Uttar Pradesh, India [2]. We chose a cut-off for negative personal attitudes when participants answered at least 3 questions with ‘probably not willing,’ or at least one question with ‘definitely not willing’ and at least one question with ‘probably not willing.’” | For clarification, the following has been added to the cut-off points explanation: “The KAP measure was used to assess the knowledge, attitudes and practices of participants regarding leprosy. On some of the questions, multiple answers were possible. A maximum score of eight could be obtained on the KAP if all correct answers are provided, even if incorrect answers were present. We defined ‘poor knowledge’ as a score of two or less out of eight, ‘moderate knowledge’ as a score between two and six and ‘adequate knowledge’ as a score of six or more on the KAP. These cut-offs were chosen arbitrarily, as no external criterion was available. The KAP measure has been used in several leprosy studies in Nepal, India, Indonesia and Brazil between 2012 and 2018 [8,26–28].” |
| Reviewer #1: No analysis plan is mentioned in the methodology | We thank the reviewer for this comment and kindly refer to methods > data management and methods > data analysis, that describe the data analysis (plan). | N/a |
| Reviewer #1: Data presented do not support the conclusions. Reviewer #2: Conclusion supported by data presented Limitations explained clearly | - | N/a |
| Reviewer #2: Comprehensive analysis clearly presented results Good quality figures | We thank the reviewer for this comment. | N/a |
| Reviewer #2: Errors found: line 280 typographical error 'martial' should be 'marital' line 325 table 5 line 240 = table 5 (duplicate number for two different tables | We apologise for these errors, they have been revised. | ‘Martial’ has been revised to ‘marital’. The final Table presented is now Table 6 instead of Table 5. |
| Reviewer #2: Clear objectives Appropriate study design for the objectives study population and sampling described in previous publication ethics = satisfactory | We thank the reviewer for this comment. | N/a |
| Reviewer #3: Noordende and colleagues have reported an important study around intervention that contributed in improving knowledge of leprosy in Uttar Pradesh, India. The study is important and adds to the current evidence. I have comments and suggestions to improve the content and presentations as below. | We thank the reviewer for his/her comments. | N/a |
| Reviewer #3: Without a schematic diagram of how study spanned over in time and the intervention, it is difficult for readers to visualize how they study might have taken place. I urge authors to present the study activities using a schematic diagram that can describe interventions, and evaluation in time period. You can change Table 2 to a figure. | This has been revised. | Table 2 is now Figure 1. |
| Reviewer #3: Regarding impact of interventions: posters and community meetings. Authors have tried to evaluate the impact of these interventions with the outcome measures as increase in knowledge and reduction in stigmatizing attitudes. First of all, somewhere it needs to be clearly acknowledged, with such interventions, and owing to the fact that they are based on pre- and post-intervention, the chances that we may over-claim the effects/impacts are high. I would generally acknowledge them by stating clearly in the limitation section about attribution versus contribution. Clearly these interventions may have contributed, but we cannot rule out other myriad factors that may have contributed to achieve the outcome | We thank the reviewer for this important comment and have added a few lines to emphasize this in our discussion. | The following has been added to the discussion > methodological considerations: “This study has several limitations. First, a randomized controlled design was not feasible given the nature of our intervention, namely, community-wide poster dissemination and meetings. Instead, we used a pre/post intervention design. It is possible that not all of the observed changes are due to the interventions – some changes may have been caused by other factors. We tried to minimize this by selecting a random sample. However, we cannot rule out other factors that may have contributed to the outcomes observed. While it is unclear how much of the effect found can be attributed to the interventions, given our findings it is very likely that the interventions have contributed to the outcomes.” |
| Reviewer #3: I think authors are describing great interventions and their impacts, but the narrow scope of discussion may limit their readability. I urge authors to expand on it more, with more literature and disciplinary terminologies, so that its implications are transferrable to other diseases and conditions. *and* Reviewer #3: While authors have very well described the interventions: posters, and community meetings. These activities, by virtue of their characteristics are included under the terminology of ‘community engagement’. Indeed, authors have clearly generated evidence around how contact intervention in the past have affected the stigma reduction, that may well be described as one of the strand or aspect of community engagement. It is essential to appreciate the term ‘community engagement’ for several reasons including, how we may have been discussing the same content but through different lenses. This also means, interventions such as ‘posters’ and ‘community meetings’ are discussed and evaluated as essential elements of community engagement in various diseases, such as malaria. Certainly, discussing these relevant literatures from other diseases will expand the scope of the current paper. This may further make other disciplinary researchers to learn from your evaluation as well. Eventually, transferability of knowledge generated in one particular disease may well be an important element for researchers working in other diseases | This has been revised. | The following has been added to the discussion: “A key feature of the interventions in the present study is that they are contextualized, relatively low-cost and easy to replicate. We ensured that the materials and messages were targeted and contextualized and that relevant topics were prioritized, e.g. cause, mode of transmission, symptoms and infectiousness of leprosy. Contextualized materials and messages are more effective than generic messages [47]. In addition, community consultation and involvement was used - this is more episodic community participation, in contrast to for example community engagement, which suggests an ongoing and active relationship [11]. ~~In addition, t~~The interventions were developed through collaborations and consultations between the target population, including persons affected by leprosy, researchers, health workers, leprosy experts, communication experts and policymakers. This maximizes the likelihood that the interventions fit with the target groups’ needs and acceptability, and the uptake of the interventions by policymakers [23].   Several successful community-based stigma reduction interventions have been conducted in the field of leprosy, all including elements of community participation or engagement, such as informing, consulting, involving, collaborating and empowering communities [10,11]*.* These studies have shown encouraging results. Successful community-based stigma reduction interventions in the field of leprosy include education and counselling through (community) stigma reduction committees [9], stigma reduction interventions through groups of health workers, volunteers and persons affected in self-help groups [8], and rights-based counselling and contact events [4]. Indeed, community participation and engagement can ensure that research is relevant and impactful. Community engagement has been successful for control and elimination of other diseases also, such as malaria [12,13]. In the present study, in addition to the community’s involvement in the development of the interventions, the community was also consulted, and discussions were held in community meetings for the purpose of education and changing negative attitudes regarding leprosy. Efforts were made to ensure community and health worker engagement in the interventions, by consultation and by involving them in preparatory meeting.” |
| Reviewer #3: I recommend authors to explain more in detail how the community meetings were held, what was the characteristic features of these meetings? Were they dialogues, monologues, presentation? Please explain the content in more details. Same for the posters, I would recommend authors to present the posters in the main manuscript, so that readers get to know what was the interventions rather than to speculate about it.  *and* Reviewer #3: Authors have stated throughout the manuscript, that contextualized messages (posters and meetings) are important. I cannot agree more, I applaud for their statement. I urge authors to illustrate such contextualization more explicitly, with more examples if possible? | We thank the reviewer for this comment. A detailed description of the the selection and development, including the pilot tests and content, of the interventions can be found as supporting information file (S1 Text). This detailed description was placed in a supporting information file to reduce the word count in the main text. We have now added S2 Text that includes the posters and an English translation. In addition, we have added more information about the community meetings in the main text. | The posters have been added as supporting information file (S2 Text):  “Two interventions are assessed in this paper: (1) posters and (2) community meetings. Posters were available in three sizes (46x58 cm, 44x14 cm and 28x23 cm) and covered the following themes: symptoms, mode of transmission, cause, curability, (free) treatment, prevention of leprosy, and inclusion of persons affected by leprosy in the community. The posters and an English translation can be found as supporting information file (S2 Text). (…)”  The following has been added to methods > interventions, to explain the meetings in more detail: “The villages in which the posters were put up were selected based on the number of leprosy patients registered at the health center since April 2014. Only villages in which at least two patients were registered were selected.” and “The villages the community meetings were held in were selected from the list of 606 villages in which posters were put up. Villages in which the Pradhan (village leader) was available on schedule meeting days and prior experience with the Pradhan was good, were selected. Community members were invited to attend the meeting through the Pradhan and by door-to-door visits from community health workers (ASHA’s). The meetings itself consisted of a short presentation about leprosy and the PEP++ project, followed by questions-and-answers and a discussion. In some meetings, two short videos about leprosy were also presented (due to technical issues this was not possible in all meetings). A health worker was present during the meetings. Meetings with key influential people were held at district or block level, while meetings with community members and persons affected by leprosy were held in the communities. Participants were also offered a leaflet with more information about leprosy (facts, myths and misconceptions) and the PEP++ project at the meetings. An overview of the reach of the two interventions can be found in Table 1. We report on perception and knowledge of leprosy before and after the interventions.” |
| Reviewer #3: There are recent suggestions/recommendations regarding the need for increase community engagement for stigma reduction strategies among community members and health care providers, authors need to acknowledge these literatures, challenge or discuss the possibilities. | This has been revised. | The following was added to methods > interventions:  “The interventions were designed based on the six steps of quality intervention development (6SQuID) [3] using a community engagement method. (…) The main input for the selection of the interventions (content and modes of delivery) came from: a) ‘leprosy perception study’ (Survey 1) of people’s knowledge and perceptions of leprosy and persons affected by leprosy [8,24,25], b) a ‘communication needs assessment’, and c) a workshop with input from persons affected by leprosy and other key stakeholders. A detailed description of the selection and development, including the pilot tests, of the interventions can be found as supporting information file (S1 Text).”  The following has been added to the introduction:  “Interventions that have reduced leprosy-related stigma include ‘contact events’ in which contact between persons affected by leprosy and community members is enhanced, socioeconomic rehabilitation, peer counselling, social marketing campaigns, community engagement interventions and mass media campaigns [4–9].” (including two references)  A paragraph about community engagement has been added to the discussion. |
