# Supplementary material for: Changing perception and improving knowledge of leprosy: An intervention study in Uttar Pradesh, India
Source: PLoS Negl Trop Dis. 2021 Aug 23;15(8):e0009654. doi: 10.1371/journal.pntd.0009654 (PMC8412405; doi:10.1371/journal.pntd.0009654)
Supplement: Attachment — Submitted filename: reviewer comments.docx [file pntd.0009654.s005.docx]

Reviewers comments

# Introduction:

Provide definitions and explain the difference between these two concepts-**perception & knowledge used in the title, also ‘attitudes’**

Methodology: Organize the text in the following order:

# Topic: Changing perception and improving knowledge of leprosy: an intervention study in Uttar Pradesh, India - may be modified how the best it suits.

# The topic is of high importance to assess the changes after intervention, scientifically not presented. To make it publishable and be useful for the readers the following major changes need to be made:

# Introduction: Provide definitions and explain the difference between these concepts in the title and used in the manuscript -perception, knowledge, attitudes and practices.

Research design:

- Study phases: Details Baseline(pre- interventional), intervention, Evaluation(post- interventional)
- Input and outcome measures

Study area: The study was conducted in two districts in Uttar Pradesh, northern India: Chandauli (population 1.95 million) and Fatehpur (population 2.63 million). All this population and districts were divided into how many cities, what are Interventional and control areas (with out control area the change can not be attributed to this intervention)

Duration of the study- For each phase in each cite

Study population: (it seems study population includes health care workers, community members, leprosy affected and close contacts) . Need to explain who are these close contacts or family members.

Data collection methods: For each of the above categories, what is Sample size, Sampling procedure used to decide sample size, methods of data collection, method of recruiting the respondents.

Methodology for intervention: What is the total no of villages in the two districts. Posters covered in 606 villages across the two districts, whether covered all the villages, or any criterial to select; Similarly 271 group meeting in how many villages and what is the criteria.

Data collection was done from a total 1067 participants in the first survey and 843 participants including from all the categories of responding. Need to clarify whether this data collection was done only from the areas ( villages/blocks) where interventions were done; if so the differences in the studied variables between pre and post intervention assessments can not be attributed to the result of this intervention. Such changes may be taking place every where over a period of time due to any other factors.

In case the data represent also certain blocks(similar) from the study area where intervention was not implemented at all, that can be the control area and changes in the interdental area are significantly higher than in the control area, the change will scientifically attributed our KAP intervention.

Clarify what is adequate knowledge and cut of points used for other variable measures.

Accordingly the manuscript may be reorganized and accordingly the summary also to be modified.
